# Screening of predicted synergistic multi-target therapies in glioblastoma identifies new treatment strategies

**DOI:** 10.1093/noajnl/vdad073

**Published:** 2023-06-13

**Authors:** Megan Houweling, Anna Giczewska, Kulsoom Abdul, Ninke Nieuwenhuis, Asli Küçükosmanoglu, Krzysztof Pastuszak, Rogier C Buijsman, Pieter Wesseling, Laurine Wedekind, David Noske, Anna Supernat, David Bailey, Colin Watts, Thomas Wurdinger, Bart A Westerman

**Affiliations:** Department of Neurosurgery, Amsterdam UMC location Vrije Universiteit Amsterdam, Boelelaan 1117, Amsterdam, The Netherlands; Cancer Center Amsterdam, Brain tumor center Amsterdam, Amsterdam, The Netherlands; WINDOW consortium, Amsterdam, The Netherlands (www.window-consortium.org); Medical University of Gdańsk, Laboratory of Translational Oncology, Intercollegiate Faculty of Biotechnology, 80-211 Gdańsk, Poland; Medical University of Gdańsk, Centre of Biostatistics and Bioinformatics Analysis, 80-211 Gdańsk, Poland; Department of Neurosurgery, Amsterdam UMC location Vrije Universiteit Amsterdam, Boelelaan 1117, Amsterdam, The Netherlands; Cancer Center Amsterdam, Brain tumor center Amsterdam, Amsterdam, The Netherlands; WINDOW consortium, Amsterdam, The Netherlands (www.window-consortium.org); Department of Neurosurgery, Amsterdam UMC location Vrije Universiteit Amsterdam, Boelelaan 1117, Amsterdam, The Netherlands; Cancer Center Amsterdam, Brain tumor center Amsterdam, Amsterdam, The Netherlands; Department of Neurosurgery, Amsterdam UMC location Vrije Universiteit Amsterdam, Boelelaan 1117, Amsterdam, The Netherlands; Cancer Center Amsterdam, Brain tumor center Amsterdam, Amsterdam, The Netherlands; WINDOW consortium, Amsterdam, The Netherlands (www.window-consortium.org); Medical University of Gdańsk, Laboratory of Translational Oncology, Intercollegiate Faculty of Biotechnology, 80-211 Gdańsk, Poland; Department of Algorithms and Systems Modelling, Faculty of Electronics, Telecommunication and Informatics, Gdańsk University of Technology, 80-233 Gdańsk, Poland; Medical University of Gdańsk, Centre of Biostatistics and Bioinformatics Analysis, 80-211 Gdańsk, Poland; NTRC Therapeutics B.V., Oss, The Netherlands; Department of Pathology, Amsterdam UMC location Vrije Universiteit Amsterdam, Boelelaan 1117, Amsterdam, The Netherlands; Princess Maxima Center for Pediatric Oncology, Laboratory for Childhood Cancer Pathology, Utrecht, The Netherlands; Department of Neurosurgery, Amsterdam UMC location Vrije Universiteit Amsterdam, Boelelaan 1117, Amsterdam, The Netherlands; Cancer Center Amsterdam, Brain tumor center Amsterdam, Amsterdam, The Netherlands; WINDOW consortium, Amsterdam, The Netherlands (www.window-consortium.org); Department of Neurosurgery, Amsterdam UMC location Vrije Universiteit Amsterdam, Boelelaan 1117, Amsterdam, The Netherlands; Cancer Center Amsterdam, Brain tumor center Amsterdam, Amsterdam, The Netherlands; Medical University of Gdańsk, Laboratory of Translational Oncology, Intercollegiate Faculty of Biotechnology, 80-211 Gdańsk, Poland; Medical University of Gdańsk, Centre of Biostatistics and Bioinformatics Analysis, 80-211 Gdańsk, Poland; IOTA Pharmaceuticals Ltd, St Johns Innovation Centre, Cowley Road, Cambridge, CB4 0WS, UK; WINDOW consortium, Amsterdam, The Netherlands (www.window-consortium.org); Institute of Cancer and Genomic Sciences, University of Birmingham, Edgbaston, Birmingham, B15 2TT, UK; WINDOW consortium, Amsterdam, The Netherlands (www.window-consortium.org); Department of Neurosurgery, Amsterdam UMC location Vrije Universiteit Amsterdam, Boelelaan 1117, Amsterdam, The Netherlands; Cancer Center Amsterdam, Brain tumor center Amsterdam, Amsterdam, The Netherlands; WINDOW consortium, Amsterdam, The Netherlands (www.window-consortium.org); Department of Neurosurgery, Amsterdam UMC location Vrije Universiteit Amsterdam, Boelelaan 1117, Amsterdam, The Netherlands; Cancer Center Amsterdam, Brain tumor center Amsterdam, Amsterdam, The Netherlands; WINDOW consortium, Amsterdam, The Netherlands (www.window-consortium.org)

**Keywords:** drug combination screen, EGFR/ERBB2/BCL2/MCL1, IDH-wildtype glioblastoma (GBM), multi-target therapy, synergistic drug combinations

## Abstract

**Background:**

IDH-wildtype glioblastoma (GBM) is a highly malignant primary brain tumor with a median survival of 15 months after standard of care, which highlights the need for improved therapy. Personalized combination therapy has shown to be successful in many other tumor types and could be beneficial for GBM patients.

**Methods:**

We performed the largest drug combination screen to date in GBM, using a high-throughput effort where we selected 90 drug combinations for their activity onto 25 patient-derived GBM cultures. 43 drug combinations were selected for interaction analysis based on their monotherapy efficacy and were tested in a short-term (3 days) as well as long-term (18 days) assay. Synergy was assessed using dose-equivalence and multiplicative survival metrics.

**Results:**

We observed a consistent synergistic interaction for 15 out of 43 drug combinations on patient-derived GBM cultures. From these combinations, 11 out of 15 drug combinations showed a longitudinal synergistic effect on GBM cultures. The highest synergies were observed in the drug combinations Lapatinib with Thapsigargin and Lapatinib with Obatoclax Mesylate, both targeting epidermal growth factor receptor and affecting the apoptosis pathway. To further elaborate on the apoptosis cascade, we investigated other, more clinically relevant, apoptosis inducers and observed a strong synergistic effect while combining Venetoclax (BCL targeting) and AZD5991 (MCL1 targeting).

**Conclusions:**

Overall, we have identified via a high-throughput drug screening several new treatment strategies for GBM. Moreover, an exceptionally strong synergistic interaction was discovered between kinase targeting and apoptosis induction which is suitable for further clinical evaluation as multi-targeted combination therapy.

**Key Points**

1. Identification of novel synergistic drug combinations via high-throughput drug screening in GBM.
2. Co-inhibition of kinases and apoptosis signaling highlights that inhibition of compensatory survival pathways form a deep vulnerability in GBM

**Importance of the Study**

Despite intensive efforts in the last decade to improve the treatment of IDH-wildtype glioblastoma (GBM) patients, recurrence of the tumor remains inevitable. Identifying effective drug combinations against GBM could improve therapeutic effects but has been difficult so far. Here we report the largest high-throughput drug combination screen to date, where we matched multi-target combinations to common genetic events as seen in patients. We identified 15 synergistic drug combinations, among those we observed exceptionally high synergy with drug combinations targeting EGFR, ERBB2, BCL2, MCL1, or Calcium ATPase. Our results implicate that simultaneous survival pathway inhibition of multiple proteins within the kinase and apoptosis pathway is needed to come to strong drug interactions. Our data provide a strong basis for further clinical assessment of the identified tailored multi-target therapy and point toward the need for pharmacokinetic assessment to translate our findings towards the clinic.

Isocitrate dehydrogenase (IDH)-wildtype glioblastoma (GBM) is a highly aggressive and heterogeneous brain tumor in adults. The standard therapy consists of a regime defined 2 decades ago: maximal surgical resection and radiotherapy combined with temozolomide.^1,2^ Acquired or inherent therapy resistance, as well as tumor cell heterogeneity, are seen as the cause of the inevitable relapse of the tumor resulting in a median survival of approximately 15 months.^3,4^ Over the last decade, extensive efforts have been made to discover new targeted therapies against GBM based on the favorable results observed in other tumor types.^5–7^ Most of these efforts have not been successful, although partial efficacy in up to 20% of patients has been seen.^8–10^ For example, Larotrectinib has shown benefits and even led to durable disease control in some patients with primary central nervous system tumors.^9,11,12^ These partial efficacies provide a starting point from which to treat larger patient populations.

As an alternative to overcome drug resistance and tackle tumor cell heterogeneity in GBM, the focus has shifted from targeted monotherapies to studying the effect of drug combination therapy.^13,14^ Such drug combination therapies can successfully overcome therapy resistance by simultaneously targeting multiple redundant or independent survival pathways, crucial for cell growth and survival.^15,16^

Assessing the effect of drug combination therapy in a preclinical setting is challenging in comparison to monotherapy. First, drug interaction measurements should be reflective of biological intracellular interactions. These interactions can have their own, non-linear dynamics, ranging from synthetic lethal, synergistic, additive to undesired antagonistic interactions.^17–19^ Second, an almost unlimited number of theoretical drug combinations necessitates the pre-selection of the most promising drug combinations.

There are two major metrics by which synergistic interactions can be assessed, either through a dose equivalence or through a multiplicative survival-based method. As yet, there is no consensus as to which method should be used given their different, sometimes mutually exclusive, interpretations of the interaction.^20–24^ Dose equivalence is based on the idea that if drug concentration A is reduced, it can be compensated by increasing drug concentration B to reach the same lethal effect.^25^ Multiplicative survival assumes that both drugs act independently where the viability effect of each drug contributes to its expected additive lethal effect.^26–28^ Since dose-equivalence and multiplicative survival are either based on multiple doses or viability at a single dose, the interpretation can lead to conflicting outcomes ranging from a drug combination that leads to lower viability without showing a dose shift to a drug combination that yields a dose shift without a noticeable viability effect. For completeness, here we use both methods in parallel to determine synergy.

Several in silico models have been developed to preselect drugs for synergy studies, including SynergyFinder 2.0 and the Cancer Drug Atlas.^29–31^ In this study, we evaluated 90 drug combinations predicted to work synergistically according to the Cancer Drug Atlas.^29^ These drug combinations were matched to personalized genetic features as they occur in GBM patients and assessed through a drug combination screen on a panel of 25 patient-derived and molecular-defined GBM cultures that reflect intra- and interpatient heterogeneity as well as tumor resistance.

The drug combination screens showed, in both short- and long-term, a high synergistic effect between agents that target kinases, like epidermal growth factor receptor (EGFR) and erythroblasts oncogene B2 (ERBB2/HER2), and agents that broadly target the apoptosis pathway including BCL2, MCL1, or Calcium ATPases. Overall, our results suggest that combined survival pathway inhibition within the kinase and apoptosis pathways is a major vulnerability of GBM which can be further exploited.

## Methods

### Frequency of Genetic Events

GBM clinical data of 393 patients present in the Cancer Genome Atlas (TCGA) was analyzed for copy number variations and mutations to assess the frequency of genetic events.^32^ Our analysis excluded gene fusions since they are very uncommon in GBM. We evaluated the frequency of amplifications, deep deletions, gains, and losses, as well as mutations in 14 genes that are frequently affected in GBM. Driver genes were selected based on their presence in the Cosmic-Cancer Mutation Census database (https://cancer.sanger.ac.uk/cosmic). For tumor suppressors, homozygous loss was considered a driving event. For patients where no apparent tumor-driving genes could be defined, drug combinations that included general cytotoxic or metabolic drugs were chosen. If no drugs were available for the target, the genes were excluded at the time of retrieving the data.

### Cell Culture and Compounds

BS153 was kindly provided by Prof. Zippelius (Beatrice Dolder Universitatsspital, Basel, Switzerland) and cultured in Dulbecco’s Modified Eagle’s Medium (DMEM, ThermoFisher Scientific, Waltham, MA, USA) supplemented with 10% Fetal Bovine Serum (Serana, Pessin, Germany) and 1% penicillin/streptomycin (ThermoFisher Scientific, Waltham, MA, USA). Glioblastoma sphere cultures (GSCs) were kindly provided by Dr. Bhat (The University of Texas MD Anderson Cancer Center, Houston, TX, USA) and Prof. Sulman (NYU Langone’s Perlmutter Cancer Center/NYU Grossman School of Medicine, New York, NY, USA), they were established via single-patient surgical resection. GBM8 was kindly provided by Dr. Bakhos Tannous (Harvard/MGH, Boston, MA, USA). Cells were cultured as described earlier.^29^ All GBM cultures were certified mycoplasma free by regular testing via http://www.microbiome.nl/.

Stock concentrations of 10 mM in 100% dimethyl sulfoxide (DMSO) were used for drug experiments, except for Bleomycin Sulfate, for which a stock concentration of 7.4 mM was used due to a lower solubility. See Supplementary Table 3 for storage and supplier information. For Drug selection and IC50 assays see Supplementary Methods.

### Drug Combination Screen Short-term

BS153, GBM8, and 23 GSCs were seeded in µClear® 384-well Flat-bottom plate 24 h before treatment. All cells were plated at a cell density of 3000 cells/well, except BS153 (2000 cells/well), GSC7-2, and GSC23 (2500 cells/well). Based on the sensitivity of each cell line, we adapted the drug range in which the drug combination was tested per cell line aiming to start around the IC50. We defined the highest tested drug concentration and from there diluted it 3-fold (Supplementary Tables 5–7). The drug combination treatment was added with the Tecan D300E dispenser. After 72 h of drug exposure, cell viability was measured with CellTiter-Glo 3D luminescent Cell Viability Assay as described above. RLUs were normalized based on DMSO control (≤0.1% DMSO in dual combinations, ≤0.5% in triple combinations). Each drug combination treatment was performed in technical duplicates and biological replicates were established for 15 drug combinations that showed synergy.

### Ethics Statement

The derivation and use of human cell lines derived from patients is covered under the approval of the institutional Biobank review board at the Amsterdam UMC location VUMC, MDACC Institutional Review Board at MD Anderson and Massachusetts General Hospital. Patient materials were obtained after routine diagnostics, coded according to the National Code for the Good Use of Patient Material, were exempt from informed consent.

## Results

### Drug Combinations Were Selected Based on Sensitivity Results

**Figure 1.**
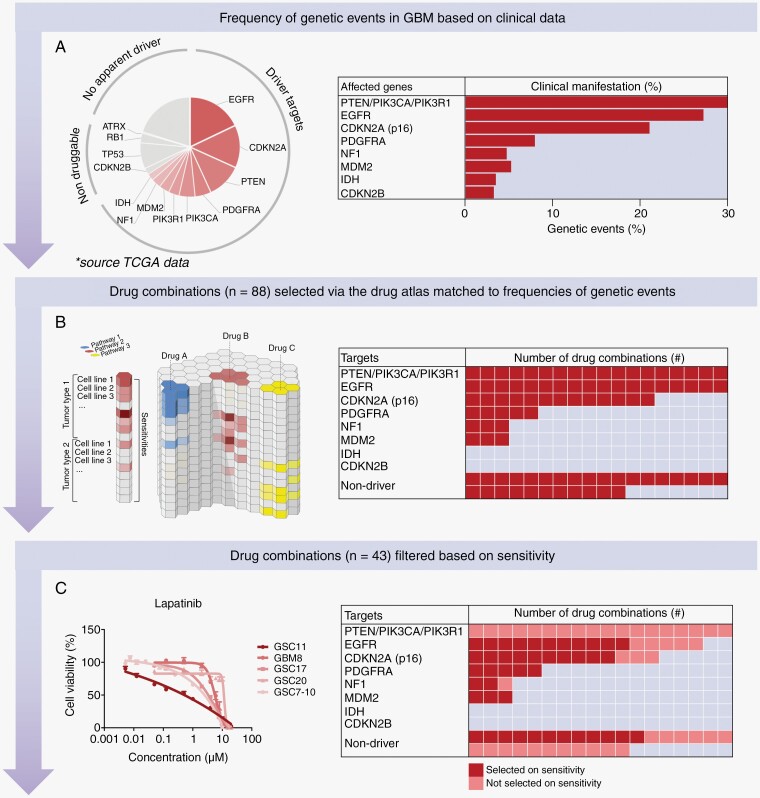
Overview of drug combination selection. (A) Pie chart showing the frequency of genetic events in GBM based on clinical data from TCGA and alongside a table with the clinical manifestation represented in percentage per driver gene. (B) Overview of the Cancer Drug atlas which was used, combined with the frequency of genetic events, to select the 90 drug combinations for driver and non-driver genes as presented in the table. Including 61 drug combinations targeting proteins of driver genes (68%) and 29 drug combinations for non-drivers (32%). (C) Dose-response curve of Lapatinib on 5 GBM cultures which was used, combined with all the other dose-response curves (see data availability, IC50 curves), to filter the 90 drug combinations based on the individual sensitivity of each monotherapy on each of the GBM cultures. The table includes the distribution of the 43 drug combinations selected, including 31 drug combinations targeting driver genes and 12 drug combinations for non-drivers.

GBM is difficult to treat due to therapy resistance and intratumoral heterogeneity, resulting in the recurrence of the tumor in patients. We envision that personalized combination therapies might lead to more effective treatment by providing more than additive (ie, synergistic) interactions. Synergy leads to stronger efficacies, may delay or even prevent therapy resistance, and avoids toxicity because lower doses can be applied. For this reason, we selected drug combinations for which the majority was matched to patients’ tumor-driving genes as commonly seen in GBM patients (frequencies are based on TCGA; *n* = 393 patients; Figure 1A).

Around 68% of GBM patients showed tumor-driving amplifications, mutations, or deep deletions in the following driver genes: EGFR, cyclin-dependent kinase inhibitor 2A (CDKN2A), Phosphatase and tensin homolog (PTEN), PDGFRA, Phosphatidylinositol-4,5-Bisphosphate 3-Kinase Catalytic Subunit Alpha (PIK3CA), Phosphoinositide-3-Kinase Regulatory Subunit 1 (PIK3R1), MDM2, Neurofibromin 1 (NF1), and IDH. IDH1 mutation (ie, R132H) was excluded since we had no models representing this mutation. Gene fusions were also excluded due to infrequent occurrences. The genes human Telomerase Reverse Transcriptase (hTERT), cyclin-dependent kinase inhibitor 2B (CDKN2B), Tumor protein P53 (TP53), Retinoblastoma 1 (RB1), and Alpha Thalassemia/Mental Retardation Syndrome X-Linked (ATRX) were excluded due to the absence of a target or the non-availability of a drug. Since CDKN2A loss leads to Cyclin-dependent kinase 4/6 (CDK4/6) activation, we included CDK4 as a substitution for CDKN2A loss. The remaining 32% of patients had no druggable genetic aberration or no clear driver gene (*n* = 124 patients).

Previously, our laboratory created a computer algorithm called the Cancer Drug Atlas, which enables us to predict which drug combinations are likely to synergistically eliminate cancer cells in a tailored way.^29^ This synergy prediction algorithm was based on Sanger GDSC1000 and Novartis/Broad CCLE drug-encyclopedia data and was applied here to predict drug combinations that match tumor-driver genes. Drug combinations were matched based on the frequency of the presence or absence of genetic events (Supplementary Tables 1 and 2). In total, 88 drug combinations were selected (Figure 1B), reflecting 45 monotherapies (Supplementary Table 3). We assessed the sensitivity of each of these monotherapies on our panel of patient-derived GBM cultures (See data availability: Supplementary Data IC50). Based on the lack of drug sensitivity, we excluded 15 monotherapies resulting in a final list of 43 drug combinations (Figure 1C and Table 1).

**Table 1.** List of 43 Drug Combinations, Including Target and Prediction Synergy Score, Selected for High-throughput Screening

Number in screen	Drug 1	Drug 2	Prediction synergy score^1^
			(0–520)
1	CGP-082996 (CDK4)	Gemcitabine (Base analogue)	503
2	CGP-082996 (CDK4)	Vinorelbine (Alkylating)	426
3	CGP-082996 (CDK4)	Obatoclax Mesylate (BCL2 and MCL1)	400
4	Lapatinib (EGFR and ERBB2)	Gemcitabine (Base analogue)	424
5	Lapatinib (EGFR and ERBB2)	Vinorelbine (Alkylating)	403
6	Lapatinib (EGFR and ERBB2)	Obatoclax Mesylate (BCL2 and MCL1)	398
7	Erlotinib (EGFR and ERBB2)	Gemcitabine (Base analogue)	420
8	Erlotinib (EGFR and ERBB2)	Vinorelbine (Alkylating)	392
9	Erlotinib (EGFR and ERBB2)	Obatoclax Mesylate (BCL2 and MCL1)	394
10	CGP-082996 (CDK4)	Thapsigargin (Ca2^+^ ATPase)	438
11	Lapatinib (EGFR and ERBB2)	Thapsigargin (Ca2^+^ ATPase)	394
12	Erlotinib (EGFR and ERBB2)	Thapsigargin (Ca2^+^ ATPase)	390
13	CGP-082996 (CDK4)	Tipifarnib (Farnesyl transferase)	395
14	CGP-082996 (CDK4)	Bleomycin (Antitumor antibiotic)	426
15	CGP-082996 (CDK4)	Etoposide (TOPO2)	433
16	CGP-082996 (CDK4)	Mitomycin C (DNA crosslinking)	432
17	CGP-082996 (CDK4)	Doxorubicin (Anthracyclines)	403
18	CGP-082996 (CDK4)	Shikonin (RSK)	404
19	Lapatinib (EGFR and ERBB2)	Tipifarnib (Farnesyl transferase)	435
20	Lapatinib (EGFR and ERBB2)	Bleomycin (Antitumor antibiotic)	395
21	Lapatinib (EGFR and ERBB2)	Shikonin (RSK)	388
22	Tipifarnib (Farnesyl transferase)	NVP-TAE684 (ALK)	514
23	Tipifarnib (Farnesyl transferase)	A-770041 (SRC)	441
24	Tipifarnib (Farnesyl transferase)	Sunitinib (PDGFR)	405
25	Tipifarnib (Farnesyl transferase)	Pazopanib (PDGFR)	424
26	Sunitinib (PDGFR)	Gemcitabine (Base analogue)	503
27	Pazopanib (PDGFR)	Gemcitabine (Base analogue)	437
28	Pazopanib (PDGFR)	BMS-536924 (HGFR)	307
29	Etoposide (TOPO2)	NVP-TAE684 (ALK)	404
30	Etoposide (TOPO2)	A-443654 (AKT 1/2/3)	404
31	RG7112 (MDM2)	Sunitinib (PDGFR)	Not calculated^2^
32	RG7112 (MDM2)	Pazopanib (PDGFR)	Not calculated^2^
33	RG7112 (MDM2)	CGP-082996 (CDK4)	Not calculated^2^
34	MG-132 (Proteaome)	Vinorelbine (Alkylating)	124
35	PHA-665752 (MET)	NVP-LAQ824 (HDAC)	147
36	Thapsigargin (Ca2^+^ ATPase)	AP-24534 (MTOR)	138
37	Thapsigargin (Ca2^+^ ATPase)	Midostaurin (KIT)	128
38	WH-4-023 (SRC)	Vinorelbine (Alkylating)	148
39	WH-4-023 (SRC)	AUY922 (HSP90)	140
40	Bleomycin (Antitumor antibiotic)	OSI-906 (IGFR)	219
41	Bleomycin (Antitumor antibiotic)	GSK269962A (ROCK)	216
42	Bleomycin (Antitumor antibiotic)	A-770041 (SRC)	135
43	Bleomycin (Antitumor antibiotic)	WH-4-023 (SRC)	131

^1^Prediciton synergy score is based on a logistic regression model, according to the model available at https://github.com/bartwesterman/drug-atlas;

^2^Predicted synergy score was not calculated due to the lack of MDM2 targeting drugs in the Cancer Drug Atlas.

### The Panel of GBM Cultures Matches Molecular GBM Characteristics

**Figure 2.**
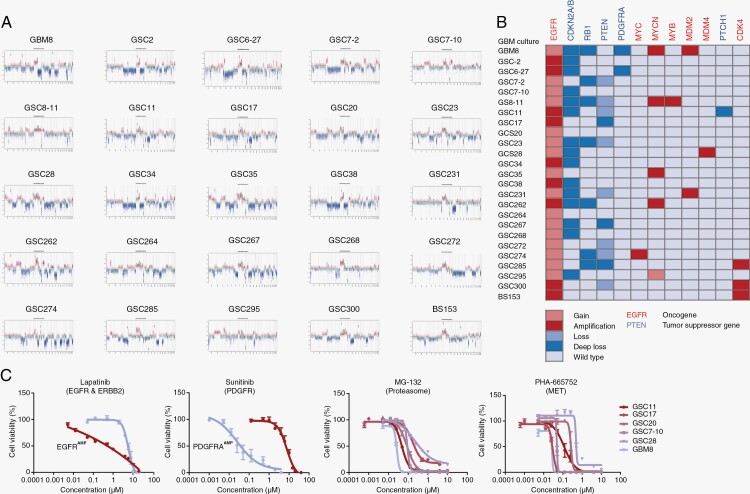
Human molecular GBM characteristics are represented in cell culture models. (A) Copy number variant (CNV) profiles of the 25 GBM cultures based on global methylation markings (EPIC array) show patterns that are representative of GBM where chromosome 7 gain and chromosome 10 loss are commonly observed. (B) Summary overview of gain/loss (light red) or amplification/deep loss (dark red) or wildtype (light blue) in interesting oncogenes (red) and tumor suppressor genes (blue) in the different GBM cultures. (C) Four dose-response curves show tumor driver as well as generalized dependencies. The dose (on the *x*-axis) and the cell viability (on the *y*-axis) of Lapatinib (EGFR targeting), Sunitinib (PDGFR targeting), MG-132 and PHA-665752 (non-targeting drugs) are shown in representative GBM cultures (*n* = 2 or *n* = 6 GBM). With Lapatinib and Sunitinib, a clear dependency on the tumor driver was seen while with non-targeting drugs like MG-132 and PHA-665752 all GBM cultures show a more or less similar sensitivity. Dose-response curves were determined in biological duplicates, each including 3 technical replicates, the data are represented as mean ± SEM. See data availability for methylation array data and all dose-response curves.

To test drug combinations, we first characterized our laboratory tumor models since GBM shows strong inter-patient heterogeneity. To mimic the clinical setting as closely as possible, we used a panel of 24 patient-derived GBM cell lines which were cultured in Neuro-Basal Medium and grew in spheroids. One serum-grown cell line, BS153, was added to the panel since it has a strong amplification of the EGFR locus. Of all the 25 cell lines a copy number variant profile was created based on the results of EPIC analysis (Figure 2A). All cell lines in the panel have a gain of chromosome 7 and therefore at least a gain of EGFR, characteristic of GBM. In 9 cell lines, we observed high-copy amplification of the EGFR locus. Chromosome 10 partial or complete loss was observed frequently as well, further supporting the panel of GBM cell cultures matching molecular characteristics of human GBMs. Homozygous loss of CDKN2A/B was seen in 15 cell lines. Other interesting aberrations in our panel of GBM cultures were seen in RB1, PTEN, PDGFRA, MYC, MDM2/4, MYB, Patched 1 (PTCH1), and CDK4 (Figure 2B).

Genetic aberrations in patient-derived GBM cultures could to some degree be matched to the drug inhibiting the protein of the respective genetic event. For instance, higher sensitivity to Lapatinib, an EGFR, and ERBB2 targeting drug, was observed for GSC11 (which is highly EGFR amplified) compared to GBM8 (EGFR gain) (Figure 2C). For the PDGFRA targeting drug Sunitinib, high sensitivity was seen for GBM8, matching its PDGFRA amplification, as compared to GSC11 for which a much lower sensitivity was seen (PDGFRA wildtype). Drugs not aimed at proteins of GBM driver genes but rather targeting mechanisms related to cell survival, such as MG-132 (Proteasome) and PHA-665752 (MET), led to a more or less similar response in all GBM cultures.

### 15 Out of 43 Dual-Drug Combinations Work Synergistically on 25 GBM Cultures

**Figure 3.**
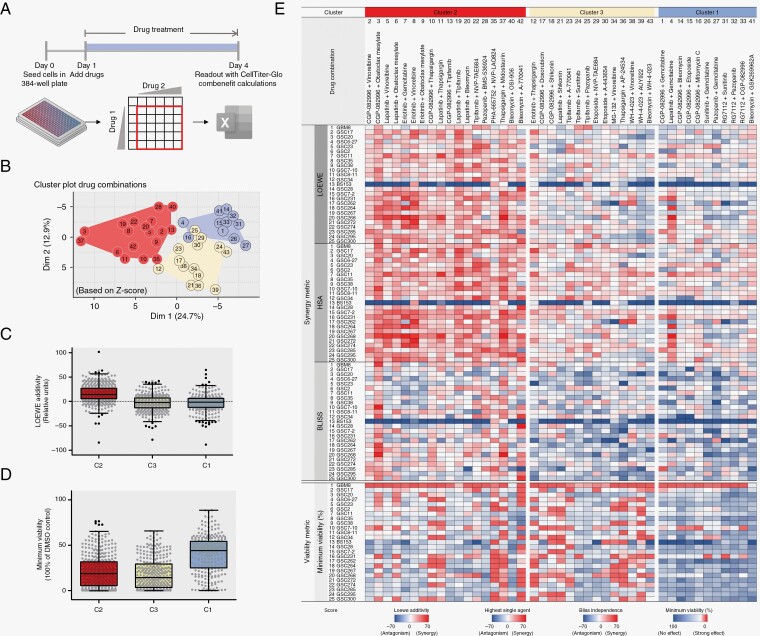
Model-based clustering of z-scale-normalized synergy and viability shows that 18 out of 43 drug combinations show a consistent drug interaction over 25 GBM cultures. (A) Schematic overview of assay set-up showing that on day 0, cells were seeded in a 384-well plate, and after 24 h, each drug combination was added via consecutive titration into a 6 × 6 matrix. On day 4, a CellTiter-Glo 3D readout was performed and cell viability results were analyzed via Combenefit. (B) Dimension reduction plot of synergy and viability results (z-scores) showing 3 clusters present in 43 drug combinations based on model-based clustering, that is, cluster 1 (blue, *n* = 11, additive with limited viability loss), cluster 2 (red, *n* = 18, synergy with viability loss), and cluster 3 (yellow, *n* = 14, additive with viability loss). (C) Mixed Scatter and Box and Whisker plot showing the raw median Loewe additivity (on the *y*-axis) for each cluster, showing a higher Loewe additivity in Cluster 2 compared to Cluster 3 and 1. (D) Scatterplot combined with a Box and Whisker plot showing a strong effect on minimum viability (on the *y*-axis) in clusters 2 and 3 compared to cluster 1. (E) Heatmap including raw synergy scores via Loewe additivity model, Highest single agent model, Bliss independence model, and the effect on minimum viability of 43 drug combinations on 25 GBM cultures ordered via the clusters retrieved via Z-score Model-based clustering. The heatmap shows a high synergy score for the 18 drug combinations included in cluster 2 (red). A high raw sum synergy score or strong effect on viability is shown in red, and antagonism or no effect on viability is shown in blue (see legend for numerical range for each synergy model). Data are average raw synergy scores (sum of synergy and antagonism) or Z-scores of 2 technical replicates. See Supplementary Table 8 for raw synergy values, see data availability for source data. Data in scatterplot/Box and Whisker plot are raw median synergy scores (*n* = 2) per drug combination (*n* = 43) of 25 GBM cultures ± Whiskers Tukey.

To assess synergy, the 43 selected drug combinations were tested on all 25 GBM cultures. Drugs were titrated in a 6 × 6 matrix in which the highest concentration was chosen such that the IC50 concentration was reached and from this concentration onwards diluted in 3-fold steps (Figure 3A and Supplementary Table 5). Given the ongoing debate in the field about whether synergy assessment should be dose or effect based, which cannot always be reconciled, we assessed drug interactions through 2 fundamentally different approaches, that is, dose equivalence or multiplicative survival, respectively.^20,22^ Firstly, we assessed synergy allowing antagonism through 2 dose-equivalence methods (the Loewe additivity model and the Highest single agent (HSA) model). We also used a multiplicative survival method (the Bliss Independence model) and assessed per drug combination the lowest tumor cell viability to determine the tumoricidal effect because synergy does not always translate to therapy efficacy. Using the Combenefit software, we assessed synergy as the sum of synergy and antagonism simultaneously. To normalize the different numerical amplitudes of the synergy metrics and viability, we normalized them using z-values, which allowed clustering to identify similarities between different synergy models. Clustering was performed via Model-based clustering, more specifically via Gaussian mixture models, which led to the optimal 3 clusters (Figure 3B). When comparing these 3 different clusters via the Loewe additivity model, Cluster 2 (Red) included drug combinations that have a higher synergy score compared to Cluster 3 (Yellow) and Cluster 1 (Blue) (Figure 3C). Furthermore, we observed a stronger tumoricidal effect in viability for the drug combinations in Cluster 2 and Cluster 3 compared to Cluster 1 (Figure 3D). We aimed to find drug combinations that consistently provide a synergistic effect as assessed through LOEWE, HSA, and Bliss in combination with a strong effect on viability. As shown in Figure 3E, Cluster 2 (Red) contains 18 combinations that met these criteria.

To validate the data independently from the previous method, we analyzed the data based on synergy only. We assessed synergy through 2 dose-equivalence methods (Loewe additivity and HSA) and 2 multiplicative survival methods (the Bliss Independence model, Chou and Talalay mutual non-exclusive method) as well as the effect on viability and performed K-mean clustering (Supplementary Figure 1). This confirmed the previous interpretation, where 15 drug combinations show consistent clustering linked to synergy and tumoricidal efficacy in both analyses. These were followed up for further study. The effects of the 15 drug combinations were reproduced independently onto the 25 GBM cultures and this led to a significantly reproducible synergy via Loewe additivity, HSA, Bliss independence, Chou and Talalay, and minimum viability (Supplementary Figure 2).

### Multiple Drug Combinations Have a Long-term Synergistic Effect on GBM Cultures

**Figure 4.**
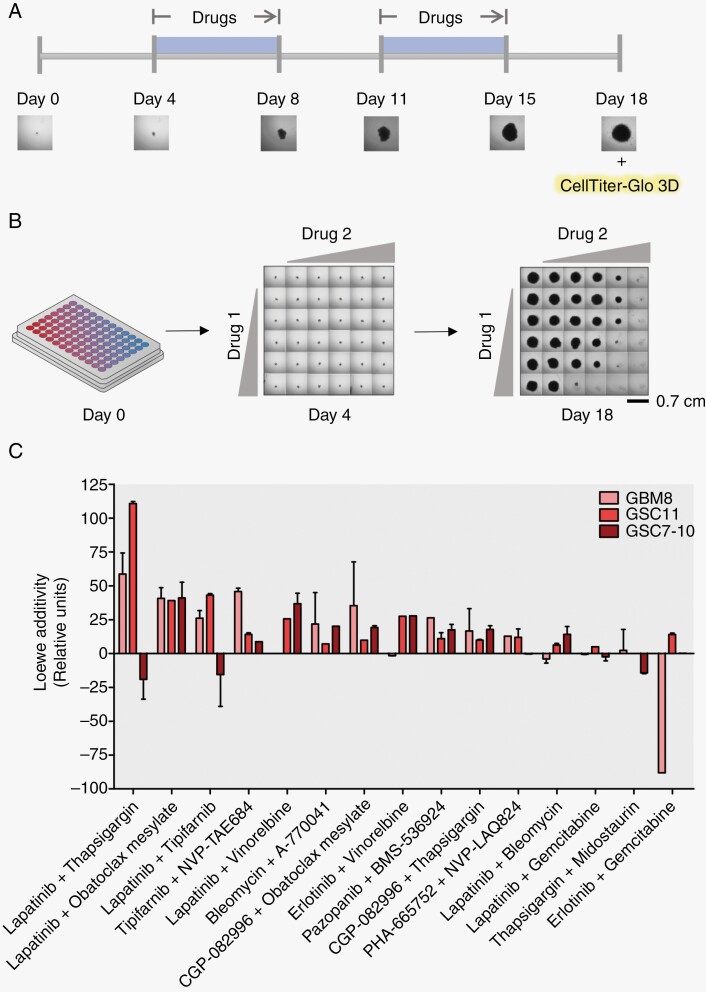
Eleven out of 15 drug combinations have a longitudinal synergistic effect on 3 GBM cultures. A. Schematic overview of the timeline for long-term screening of synergy in GBM8 (PDGFRA amplified), GSC11 (EGFR amplified), and GSC7-10 (EGFR gain) showing that spheroids were twice exposed to drug combination treatment for 5 consecutive days and were imaged 6 times to assess the growth. On day 18, cell viability was assessed via CellTiter-Glo 3D. (B) Overview of the single sphere assay where 750 cells/well were seeded in a round bottom 96-well plate to allow spheroid growth, drug treatment was titrated into the 6 × 6 matrix which was followed via imaging up on day 4 to 18, every 3 to 4 days. See legend for spheroid size. (C) Histogram showing the synergy scores according to Loewe additivity model (*y*-axis) for 15 drug combinations on 3 GBM cultures. For spheroid phase-contrast images and cell viability see data availability. See Supplementary Table 11 for synergy values.

To be able to translate these combinations to the clinic, we investigated whether synergy remains stable over a longer time frame. This had to be restricted to 3 GBM cultures for practical reasons. We choose 3 GBM cultures representative of the major genetic profiles as they occur in patients, GBM8 (PDGFRA/MYCN amplified), GSC11 (EGFR amplified), and GSC7-10 (EGFR gain). These 3 cultures were plated in a 96-well plate to form a single spheroid and were exposed in a time frame of 18 days twice for 5 consecutive days to the same drug combinations. We followed the spheroid growth via imaging each well and the cell viability was assessed using CellTiter-Glo 3D on day 18 to calculate synergy with antagonism included (Figure 4A, B). The longitudinal synergy effect of the 15 drug combinations is ranked based on Loew additivity (Figure 4C). The highest-ranked Loewe additivity was observed with Lapatinib and Thapsigargin in GSC11, an EGFR-amplified cell line. Lapatinib in combination with Obatoclax Mesylate showed a consistently high synergy in all 3 GBM cultures, only linked to EGFR amplification in synergy-only conditions (Figure 4C, Supplementary Figure 3). Overall, 11 out of 15 drug combinations lead to a longitudinal synergistic effect.

Furthermore, we compared the reproducibility of the technical and biological synergy determined via the Loewe additivity model. The technical replicates were significantly similar (Supplementary Figure 4A). Biological reproducibility was difficult to establish (Supplementary Figure 4B).

### Targeting EGFR and Apoptosis is a Generalized Mechanism of Action Leading to Synergy

Through the longitudinal synergy assessment in GBM cultures, we observed that targeting EGFR and apoptosis is a generalized mechanism that gives a prolonged response. Lapatinib in combination with Obatoclax mesylate and in combination with Thapsigargin gave a high synergistic effect. Obatoclax Mesylate is a broad BCL-targeting drug and Thapsigargin is an inhibitor of calcium ATPase. As follow up we investigated the relevance of apoptosis signaling in GBM with a focus on drugs that would be applicable in the clinic.

**Figure 5.**
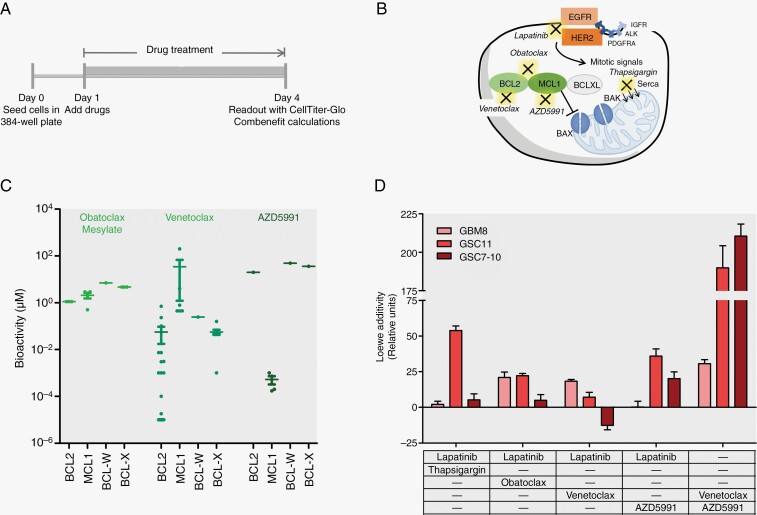
Discovery of new synergistic combination therapy including Venetoclax (BCL2) and AZD5991 (MCL1). (A) Schematic overview of assay setup. GBM8, GSC11, or GSC7-10 were seeded in 384-well plates and 24 h after seeding, drug combinations were added as previously described. When 3 drugs were used, the titration of the third drug was performed over replicate plates where the 2 other drugs were titrated on. On day 4, a readout with CellTiter-Glo 3D was performed and synergy (ie, sum of synergy and antagonism) was calculated via Combenefit. (B) Schematic depiction of pathways targeted by the different drugs. Obatoclax Mesylate is a broad BCL-family targeting drug while Venetoclax is specific for BCL2 and AZD5991 is specific for MCL1. Lapatinib targets both EGFR and HER2 (ERBB2). (C) Scatterplot showing the IC50 bioactivity of Obatoclax Mesylate, Venetoclax and AZD5991 in μM concentrations. Curated data was obtained from CHEMBL as well as from Tron et al.^39^ (D) Histogram showing the synergy scores according to Loewe additivity model (*y*-axis) for Lapatinib combined with Thapsigargin, Lapatinib combined with Obatoclax Mesylate, Lapatinib combined with Venetoclax, Lapatinib combined with AZD5991 and Venetoclax combined with AZD5991 on GBM8 (light red), GSC11 (red), and GSC7-10 (dark red). Data are shown as mean ± SEM.

To improve the translatability to the clinic, we searched for a replacement for Obatoclax Mesylate that targets the same proteins. Obatoclax Mesylate is defined as a broad BCL-targeting drug, including BCL2 and MCL1, but with a low specificity in a micromolar range. From the available MCL1 and BCL2 targeting drugs, Venetoclax and AZD5991 have high potency for their respective targets compared to Obatoclax Mesylate (Figure 5B, C). Based on these characteristics, we tested Lapatinib in combination with Venetoclax (BCL2); Lapatinib in combination with AZD5991 (MCL1); Venetoclax in combination with AZD5991 (Figure 5A).

Lapatinib in combination with either Venetoclax or AZD5991 led to similar synergies as observed with Lapatinib in combination with Thapsigargin or Obatoclax Mesylate (Figure 5D, Supplementary Figure 5). Interestingly, Venetoclax with AZD5991 showed to have a compelling high dose-equivalence score compared to the other dual drug combinations. Hence highlighting the redundancy of the BCL2 and MCL1 targets.

Together, these results show that from a prioritized set of 43 drug combinations, 11 drug combinations have a long-term synergistic effect on GBM. Furthermore, the apoptosis pathway showed to be one of the mechanisms involved in this long-term effect in GBM cells.

## Discussion

In recent years, targeted therapy has been explored unsuccessfully in clinical trials for GBM.^6^ Moreover, high-throughput tailored drug combination screens have yet to be performed in GBM. This is an important omission, since synergistic combination therapy may allow lower dosing, favoring an optimal efficacy versus adverse events toxicity ratio in these patients and overcome therapy resistance.

The prediction of synergistic drug combinations is very challenging due to the enormous complexity of possible interactions that might each be dose dependent. Here, we have performed the largest tailored drug combination screen to date in GBM, using a high-throughput effort where we selected 90 drug combinations for their activity in 25 patient-derived GBM cultures. We prioritized drug combinations based on our previously established in silico model called the Cancer Drug Atlas,^29^ matched them to the frequency of genetic driver events in GBM patients and then selected 43 drug combinations for further evaluation based on the in vitro monotherapy effect. From these, 15 combinations showed a consistent synergistic as well as a tumoricidal effect over 25 GBM cultures. This consistent pattern was identified through a novel synergy evaluation method where z-scale normalized synergy and viability values were clustered, consolidating dose equivalence, multiplicative survival as well as total survival. We subsequently tested the synergistic effect of the selected 15 drug combinations in a long-term assay (ie, 18 days of drug exposure), to identify which combinations provide a prolonged effect without being overtaken by therapy resistance. In total 11 out of 15 drug combinations showed a longitudinal synergistic effect. Together, these data form a novel and extensive resource for drug combination therapy in GBM, which includes several synergistic combinations that could be interesting for further study.

Lapatinib with Thapsigargin and Lapatinib with Obatoclax Mesylate showed to be the top-ranked synergistic combinations in GBM cultures in the long-term assay. Both combinations target the kinase EGFR and the apoptosis pathway. The discovery of targeting the oncogenic driver EGFR and its redundant partner ERBB2, in combination with dual BCL-family targeting, forms a new strategy against GBM. It is not only a targeted therapy but tailored as well for tumors with an EGFR amplification since the most significant synergistic effect was seen in GSC11 cells in which EGFR is amplified. A similar synergistic effect can be expected in other tumors with an EGFR gain or amplification. In contrast, a tumor culture that relies more on PDGFRA for cell survival and growth, such as GBM8, may be less responsive to this combination therapy.

The potency of the drugs is very important for the translatability of this new treatment strategy. We, therefore, interrogated the apoptosis pathway further by replacing Obatoclax Mesylate with Venetoclax (BCL2 inhibitor) or AZD5991 (MCL1 inhibitor) which targets the apoptosis pathway as well but with higher sensitivity compared to Obatoclax Mesylate. Venetoclax and AZD5991 both act in a nanomolar range. Interestingly, the combination of Venetoclax and AZD5991 on its own led to a high synergy score in GBM. This highlights that targeting multiple apoptosis-related proteins on itself already leads to strong synergistic effects.

Furthermore, the pharmacokinetics and toxicity of the drugs and their combination play an important role as well. In order to test the identified combinations in mice to assess their translatability to the clinic, several considerations should be taken into account. First, the pharmacokinetics of drugs and their combination should be aligned such that cells are affected simultaneously at sufficient doses. Therefore, a pharmacokinetic study to measure free drug concentrations in the brain will indicate the feasibility of the approach. Lapatinib has shown to be well tolerated in phase I/II studies, but due to inadequate delivery into the brain, might not be the best-suited inhibitor.^33^ Other dual kinase inhibitors, such as AZD3759, might be better suitable for future studies.^34^ Regarding toxicity, Venetoclax treatment can be associated with tumor lysis syndrome, although several clinical studies do show a safe long-term continuous use of Venetoclax in chronic Lymphocytic Leukemia and non-Hodgkin Lymphoma.^35–37^ Furthermore, MCL-1 inhibitors are associated with cardiovascular toxicities, which might be enhanced in combination with Venetoclax.^38^ Therefore, close health monitoring is needed when combining these drugs. For Venetoclax and AZD5991 the blood-brain barrier penetration is unknown.

In order to test interactions of drugs in mice, an assessment of the contribution of each drug to the sum of effects is required to validate the composite effect between all drugs. The interaction can be shown through effect based (ie, multiplicative survival) or potency based (ie, dose equivalence) methods, although the latter require many more mice because multiple doses will have to be evaluated. The multiplicate method using doses close to the maximal tolerable dose followed by a statistical assessment based on Bliss independence would be the most straightforward method.

In conclusion, by using an unbiased computational prioritization coupled with high-throughput screening, we have identified a striking new treatment regime for GBM. Long-term synergy was found when co-inhibiting kinases and apoptosis signaling which highlights that inhibition of compensatory survival pathways form a deep vulnerability in this tumor type. Research into the pharmacokinetics, including blood-brain barrier penetration and target engagement of the identified drugs, as well as their combinations, or possible new candidate drugs for the identified targets are now needed to pave the way for translation towards the clinic.

## Supplementary Material

vdad073_suppl_Supplementary_Data

vdad073_suppl_Supplementary_Figures

## Data Availability

All IC50, raw synergy data, spheroid images, model-based clustering and K-Means clustering are available on: https://github.com/bartwesterman/Houweling.
