# Supplementary material for: Screening of predicted synergistic multi-target therapies in glioblastoma identifies new treatment strategies
Source: Neurooncol Adv. 2023 Jun 13;5(1):vdad073. doi: 10.1093/noajnl/vdad073 (PMC10347974; doi:10.1093/noajnl/vdad073)
Supplement: vdad073_suppl_Supplementary_Data [file vdad073_suppl_supplementary_data.docx]

**Supplementary methods**

**IC50 assay**

IC50s were established on BS153, GBM8, and 23 patient-derived GSCs as previously described ^1, 2^. Single-cell suspensions were obtained using Accutase as described above. In short, 3000 cells/well were seeded in µClear® 384-well Flat-bottom plate 24h before treatment (#781097, Greiner Bio-one, Kremsmünster, Austria). GSC7-2 and GSC23 were seeded at 2500 cells/well, and BS153 at 2000 cells/well. Initial drug concentrations were estimated based on an independent assay (data not shown), followed by precise titration centered around the IC50. Drugs were added one day after plating with the Tecan D300E dispenser (Tecan Group Ltd, Männedorf, Switzerland). After 72h of drug exposure, cell viability was measured with CellTiter-Glo 3D luminescent Cell Viability Assay (Promega, Madison, WI, USA) according to the manufacturer’s protocol. The relative light units (RLUs) were measured with a Tecan Infinite® 200 reader using iControl 1.10 software, RLUs were always normalized based on DMSO controls. The assays were performed as technical triplicates and biological replicates. If a drug showed a sensitivity above 5 μM or if a common aberrant dose-response curve was seen in the cell lines (i.e. no response at all or responses that fluctuated consecutively positively and negatively), the drug was excluded from further screening. The cell titer glo assay was independently confirmed using a crystal violet staining (see **Supplementary Figure 6**).

**Drug combination selection**

Based on the frequency of mutations and CNVs in the different driver and non-driver genes, we set out to test 90 drug combinations, of which 61 target proteins of driver genes and 29 targeting proteins of non-driver genes or mechanisms (Supplementary table 1). The drug combinations were selected through prioritization of the Cancer Drug Atlas; a database created previously by our group that ranks specified drug combinations that are likely to interact synergistically based on publicly available IC50 drug sensitivity data and CNV/mutation data of 40 serum-grown CNS-cell lines ^3^. The presence of the genetic and drug sensitivity data allowed the matching of predicted combination therapies to at least one tumor-driving genetic event, making it possible to select drug combinations that target proteins of driver and non-driver genes of interest. To select the drug combination for driver genes, we ranked the top 20 drug combinations per CNS cell line, based on the synergy prediction. These top 20 ranked drug combinations per cell line were merged into one dataset according to their overall predicted synergy score. Per driver gene, the highest-ranked drug combinations were selected. Although mouse double minute 2 homolog (MDM2)-targeting drugs were not present in the data that was used to create the Cancer Drug Atlas, the relatively frequent amplification of MDM2 in GBM patients made us decide to include three combinations that targeted MDM2 using the RG7112 drug. Since IDH mutations are not commonly seen in high-grade gliomas and no IDH-targeting drug was present in the Cancer Drug Atlas, we did not include combinations that would target IDH, altogether resulting in a total of 59 drug combinations targeting driver genes ^4^.

From the 40 GBM cell lines in the drug atlas, 11 CNS cell lines did not contain obvious aberrations in the driver genes of interest and were therefore used to select drug combinations matched to the frequency of non-driver genes. For each of these 11 cell lines, the 20 drug combinations with the highest predicted synergy score were retrieved from the Cancer Drug Atlas. The top 20 drug combinations in M059J had a high synergy score compared to the other 10 cell lines. Therefore, we decided to include 9 drug combinations from M059J. From the remaining 10 cell lines, we included the two highest predicted synergistic drug combinations. If a drug combination was already selected via a different route or the drug was not available for preclinical research (e.g. Bryostatin 1), we chose the following combinations in the ranked list. In total, 88 drug combinations were included; see Supplementary Table 2.

**Methylation profiling**

We isolated genomic DNA from all GBM cultures, including BS153, with DNeasy Blood and Tissue kit (Qiagen, Hilden, Germany). We optimized the manufacturer’s protocol by adding 30 μL RNase mixture, containing 2 μL RNase A (10 mg/mL), 2 μL MgCl_2_, 3 μL PCR buffer (10x), and 23 μL sterile water, onto the column after the first wash and incubated for 10 min at room temperature to remove contaminating nucleic acids to obtain purified genomic DNA. DNA quantity and purity were estimated on a Nanodrop 8000 Spectrophotometer (ThermoFisher) according to protocol. DNA with a purity of A_260/280_ between 1.7-2 was considered sufficiently pure to proceed with methylation profiling. Methylation profiling was performed via standard procedure with the Infinium Methylation EPIC BeadChip Kit by the Human Genomics Facility of the Genetic Laboratory of the Department of Internal Medicine (Erasmus Medical Center, Rotterdam, the Netherlands). We used the publicly available methylation-based classifier of central nervous system tumors (German Cancer Research Center (DKFZ) and Heidelberg University, Heidelberg, Germany; https://www.molecularneuropathology.org/mnp/,^5^) for creating CNV profiles and epigenetic characterization of our GBM cultures and to match this information to previously obtained data ^6^.

**Drug combination screen long-term**

GBM8, GSC11, and GSC7-10 exhibit platelet-derived growth factor receptor A (PDGFRA), Epidermal growth factor receptor (EGFR) amplification, and chromosome 7 gain, respectively, therefore representing heterogeneous genetic features that differ between GBM patients and were therefore selected for drug combination screening over a longer period. Cell cultures were seeded at a cell density of 750 cells/well in a 96-well, cell-repellent round bottom Cellstar® plate (Greiner, Alphen aan den Rijn, Netherlands). On days 1, 4, 8, 11, 15, and 18 spheroid phase-contrast images were automatically taken with a Leica DMI3000 microscope (Leica, Rijswijk, the Netherlands). After images were taken on day 4 and day 11, the NBM medium was refreshed by the removal of 50 μL and adding fresh NBM medium. On both days followed by drug combination treatment as described above, for drug concentrations and ranges see Supplementary Tables 5 and 10. On days 8 and 15, the NBM medium was maximally refreshed to remove drug treatment and thereby establish a recovery period. On day 18, cell viability was measured by CellTiter-Glo 3D luminescent Cell Viability Assay as described above to assess the effect of drug combination treatment. RLUs were normalized based on DMSO control (≤0,1% DMSO), and each drug combination treatment was performed in technical duplicates. Biological replicates were established for 6 drug combinations.

**Clustering of synergy and viability data**

To be able to perform clustering based on all synergy models combined, we normalized the raw synergy scores to a Z-score^7^. Normalization was performed by determining the average synergy score and standard deviation of the 43 drug combinations on the 25 cell lines per model. Technical replicates were analyzed separately. Before performing model-based clustering, data was properly scaled and cluster tendencies were assessed using Hopkins statistics and visually using principal component analysis (PCA). Hopkins statistics (H=0.62) indicated that data is clusterable with no strong separation between clusters. As a result of applying the elbow method to a Screen plot obtained from performed PCA, we concluded that our data should be presented in three-dimensional space. After assessing clustering tendencies, model-based clustering was performed using BIC as a method for best method selection. The spherical model with equal volume (EII), and with three optimal clusters was selected as the best method. Subsequently, the separation between clusters was assessed using uncertain plots two- and three-dimensional space indicating the proper assignment of clusters. Furthermore, we assessed the probabilities of cluster membership that showed good separation between clusters. See data availability for data source and script.

To validate the clustering method independently, we analyzed the interaction between drugs by using K-Means clustering onto synergy values only (excluding the contribution antagonism) according to the following script: https://afit-r.github.io/kmeans_clustering. The K-Means clustering includes synergy and effect on viability based on the four models mentioned above but also included the mutual non-exclusive Chou and Talalay multiplicative survival method as described previously ^3^. See data availability for data source and script.

**Data analysis of synergy using z-scales followed by clustering**

The interaction of two drugs can be analyzed via different approaches based on dose-equivalent models and/or multiplicative models. Dose-equivalent models include Loewe additivity and the Highest Single Agent (HSA). While multiplicative models include Bliss’s independence model and the non-exclusive Chou and Talalay model ^8-11^. Finally, the effect on viability is another aspect that can be incorporated into analyzing the interaction of two drugs. In the field, there is no consensus reached as to the most reliable model for calculating synergy ^12, 13^. We, therefore, analyzed the sum synergy, synergistic and antagonistic interactions, via two dose-equivalent models (Loewe and HSA), multiplicative survival (Bliss) and the total viability effect. To calculate synergy, we used the Combenefit package, a software that allows automatic model-based analysis, to calculate synergy based on Loewe additivity (dose-equivalent), Highest single agent (dose-equivalent), and Bliss independence (effect model) ^14^. Combenefit software is freely available at https://sourceforge.net/projects/combenefit/. We displayed the 6x6 viability data in a predefined .xls format to be provided in batch to the Combenefit software (See data availability, Supplementary data Combenefit). Data were excluded if a data point was missing in the monotherapies and a missing data point in the drug combination treated well was constituted with 100% viability allowing calculation of synergy. Aside from assessing the drug interaction based on synergy, we incorporated the effect on viability as well to determine the tumoricidal effect because synergy does not always translate to therapy efficacy. We used for each drug combination the lowest viability reached in the 5x5 matrix. Technical replicates were analyzed separately from each other and an average synergy score was determined (Supplementary Tables 8, 9, 11, and 12). Biological replicate synergy scores were analyzed as well (see data availability and Supplementary Table 13). Drug combination synergy scores above zero up to 300 indicate synergy ^15^. All synergy values (three metrics) and viabilities (one metric) were normalized to z-values on which clustering was performed to identify groups of effective drug combinations. Details regarding the clustering is provided in the supplementary methods.

**Statistics**

All statistical analyses were performed using Graphpad Prism 5 software and Excel.

**References**

1. Brahm CG, Abdul UK, Houweling M, et al. Data-driven prioritization and preclinical evaluation of therapeutic targets in glioblastoma. Neurooncol Adv 2020;2:vdaa151.

2. Houweling M, Abdul UK, Brahm C, et al. Radio-sensitizing effect of MEK inhibition in glioblastoma in vitro and in vivo. J Cancer Res Clin Oncol 2023;149:297-305.

3. Narayan RS, Molenaar P, Teng J, et al. A cancer drug atlas enables synergistic targeting of independent drug vulnerabilities. Nature Communications 2020;11:2935.

4. Yan H, Parsons DW, Jin G, et al. IDH1 and IDH2 mutations in gliomas. New England journal of medicine 2009;360:765-773.

5. Capper D, Jones DTW, Sill M, et al. DNA methylation-based classification of central nervous system tumours. Nature 2018;555:469-474.

6. Bhat KPL, Balasubramaniyan V, Vaillant B, et al. Mesenchymal differentiation mediated by NF-κB promotes radiation resistance in glioblastoma. Cancer Cell 2013;24:331-346.

7. Kreyszig E. Advanced Engineering Mathematics Wiley, 1979.

8. Chou T-C, Talalay P. Analysis of combined drug effects: a new look at a very old problem. Trends in Pharmacological Sciences 1983;4:450-454.

9. Chou TC, Talalay P. A simple generalized equation for the analysis of multiple inhibitions of Michaelis-Menten kinetic systems. J Biol Chem 1977;252:6438-6442.

10. Chou TC, Talalay P. Generalized equations for the analysis of inhibitions of Michaelis-Menten and higher-order kinetic systems with two or more mutually exclusive and nonexclusive inhibitors. Eur J Biochem 1981;115:207-216.

11. Schindler M. Theory of synergistic effects: Hill-type response surfaces as 'null-interaction' models for mixtures. Theor Biol Med Model 2017;14:15.

12. Foucquier J, Guedj M. Analysis of drug combinations: current methodological landscape. Pharmacol Res Perspect 2015;3:e00149.

13. Twarog NR, Connelly M, Shelat AA. A critical evaluation of methods to interpret drug combinations. Scientific Reports 2020;10:5144.

14. Di Veroli GY, Fornari C, Wang D, et al. Combenefit: an interactive platform for the analysis and visualization of drug combinations. Bioinformatics 2016;32:2866-2868.

15. Menden MP, Wang D, Mason MJ, et al. Community assessment to advance computational prediction of cancer drug combinations in a pharmacogenomic screen. Nature communications 2019;10:1-17.
