# Supplementary material for: Screening of predicted synergistic multi-target therapies in glioblastoma identifies new treatment strategies
Source: Neurooncol Adv. 2023 Jun 13;5(1):vdad073. doi: 10.1093/noajnl/vdad073 (PMC10347974; doi:10.1093/noajnl/vdad073)
Supplement: vdad073_suppl_Supplementary_Figures [file vdad073_suppl_supplementary_figures.docx]

**Houweling et al. Supplementary Information Index**

Contents

[**Supplementary Figure 1. K-means clustering of z-scale-normalized synergy and viability identifies consistent drug interactions over 25 GBM cultures.** 2](#_Toc132750157)

**Supplementary Figure 2. Synergy and effect on minimum viability are reproduced consistently in biological replicates in the short-term assessment**………………………..4

[**Supplementary Figure 3. Independent analysis based on synergy only ranked Lapatinib combined with Obatoclax Mesylate as the highest synergistic combination on three GBM cultures over 18 days.** 5](#_Toc132750158)

[**Supplementary Figure 4. Synergy in long-term assessment can be reproduced in technical replicates but is difficult for biological replicates.** 6](#_Toc132750159)

[**Supplementary Figure 5. Viability heatmaps of the dual combinations including Lapatinib, Thapsigargin, Obatoclax Mesylate, Venetoclax and AZD5991.** 7](#_Toc132750160)

[**Supplementary Figure 6. Viability measurements based on crystal violet (total protein/DNA staining) match with cell titer glo (ATP based) measurement in most cases.** 8](#_Toc132750161)

**Supplementary Tables**

<https://www.dropbox.com/s/9hzxtubqorfwbkc/Supplementary%20tables_Houweling%20et%20al_30Oct.xlsx?dl=0>

**Houweling et al. Supplementary Figures**


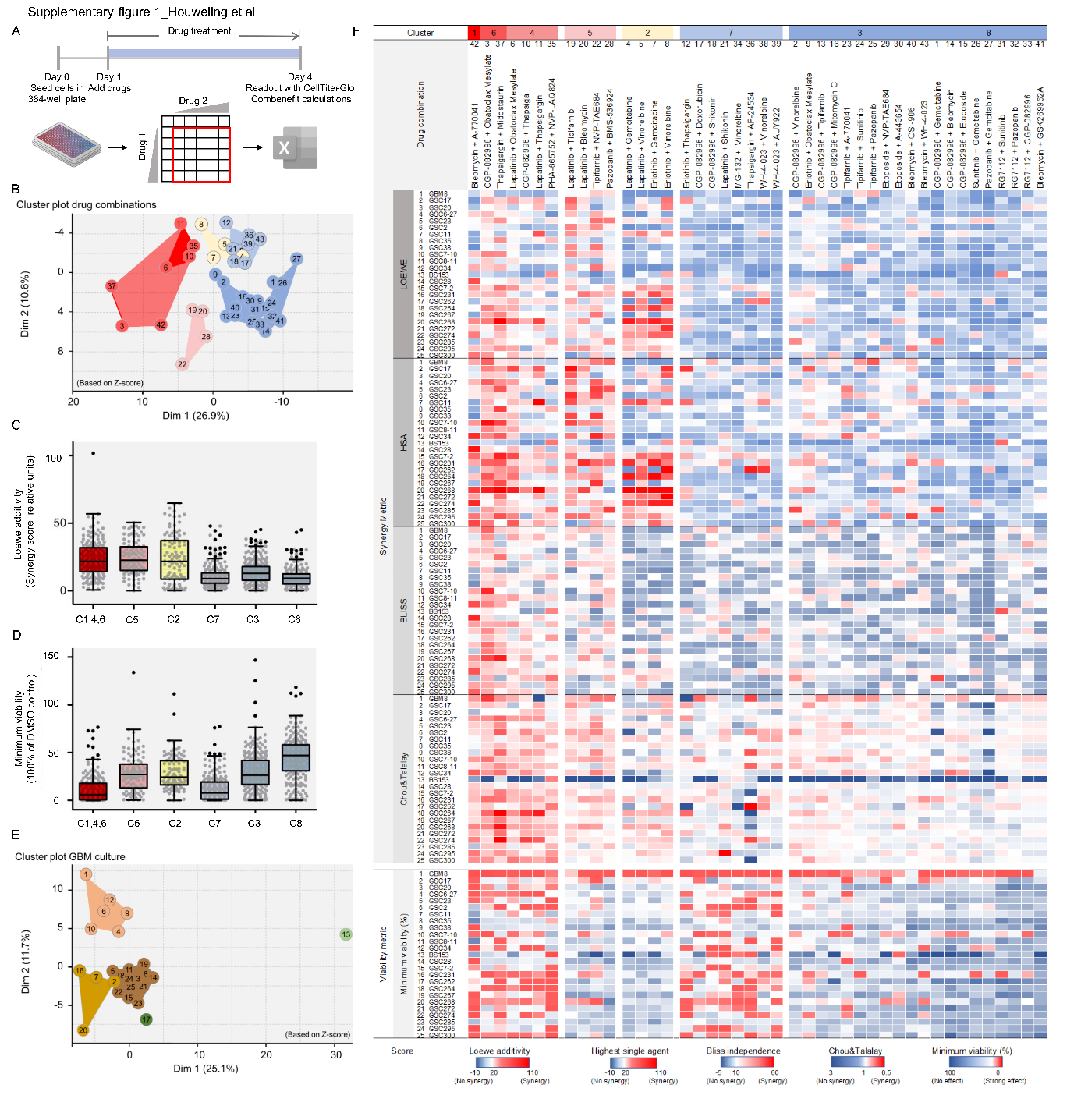


**Supplementary Figure 1. K-means clustering of z-scale-normalized synergy and viability identifies consistent drug interactions over 25 GBM cultures.** A. Schematic overview of assay set-up showing seeding of cells in a 384-well plate on day 0, followed by drug combination treatment via titration into a 6x6 matrix after 24h. On day 4, CellTiter-Glo 3D readout was performed and cell viability results were analyzed via Combenefit. B. Principal component plot of the 43 drug combinations based on the z-scores. The individual clusters based on K-means clustering are shown. C. Mixed Scatter and Box and Whisker plot showing the raw median Loewe additivity (on the y-axis) per cluster group, showing a higher Loewe additivity in the combined group (including Cluster 1,4,6), Cluster 5, and Cluster 2 compared to Clusters 3 and 8. D. The plot shows a strong effect on minimum viability (on the y-axis) in the group (including Clusters 1, 4, and 6) and Cluster 7. Data shown are raw median synergy scores (n=2) per drug combination (n=43) of 25 GBM cultures ± Whiskers Tukey. E. Principal component plot of the average synergy score of the 43 drug combinations in each of the 25 GBM cultures. The cluster plot shows the deviating response of BS153 (13 (green), DMEM with FCS cultured) compared to the other 24 GBM cultures (NBM cultured). F. Heatmap including raw synergy scores via Loewe additivity model, Highest single agent model, Bliss independence model, Chou and Talalay, and the effect on minimum viability of 43 drug combinations on 25 GBM cultures ordered via the clusters retrieved via Z-score k- mean clustering. The heatmap shows a high synergy score for 15 drug combinations, comprehended in Clusters 1,4,6 (Red), 5 (Light red), and 2 (Yellow, subset of GBM cultures), on the GBM cultures. Data are average raw synergy scores or Z-scores of two technical replicates. A high raw sum synergy score or strong effect on viability is shown in red, and no synergy or no effect on viability is shown in blue (see legend for numerical range for each synergy model). See Supplementary Table 9 for raw synergy values and see data availability for the data source.


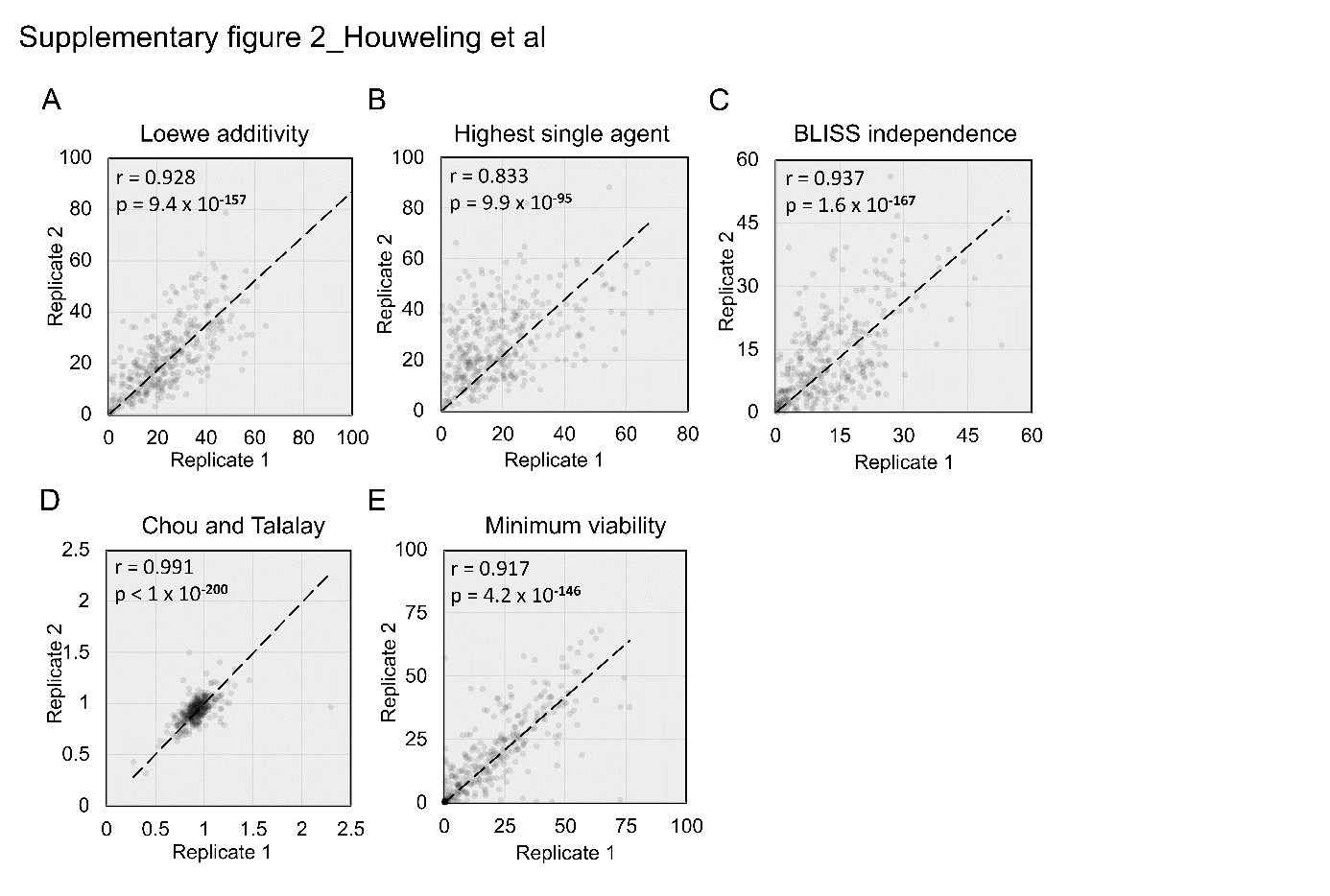
**Supplementary Figure 2. Synergy and effect on minimum viability are reproduced consistently in biological replicates in the short-term assessment.** A. Scatterplot of synergy determined via Loewe additivity model. B. Highest single agent model. C. Bliss independence model. D. Chou & Talalay. E. Minimum viability. The data included in all scatterplots are the average (of technical replicates) raw synergy score of replicate 1 vs. replicate 2. P-value was calculated using the regression coefficient and number of sampling (n=362) resulting in a t-factor. See data availability for source data.

**
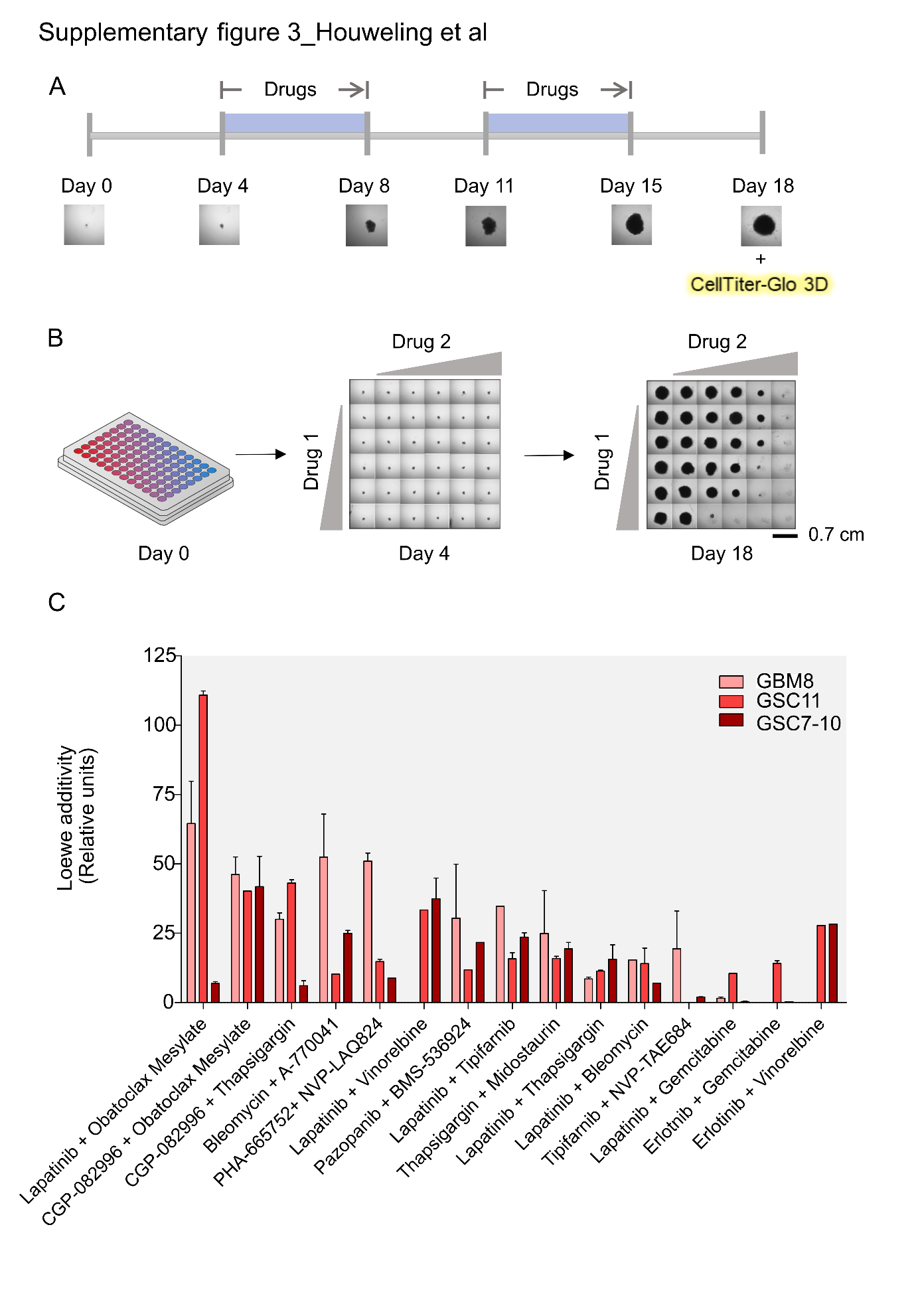
**

**Supplementary Figure 3. Independent analysis based on synergy only ranked Lapatinib combined with Obatoclax Mesylate as the highest synergistic combination on three GBM cultures over 18 days.** A and B. Schematic overview of performed long-term drug combination assay in which synergy only was assessed at day 18 via cell viability readout with CellTiter-Glo 3D. See legend for spheroid size. (See Figure 3 for detailed assay information). C. Histograms of synergy scores (here excluding the antagonistic component, determined via Loewe additivity model) for 15 drug combinations assessed on GBM8 (PDGFR amplified), GSC11 (EGFR amplified), and GSC7-10 (EGFR gain). For spheroid phase-contrast images see data availability. For cell viability see Supplementary Figure 5. See Supplementary Table 12 for synergy values.


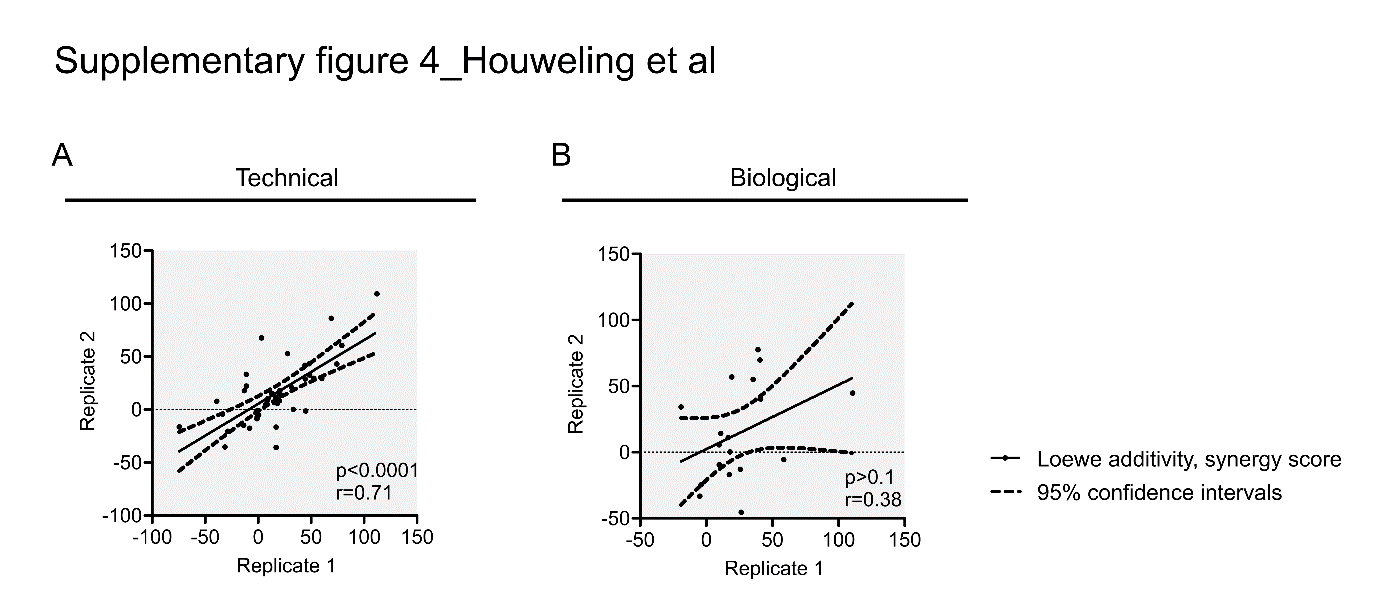


**Supplementary Figure 4. Synergy in long-term assessment can be reproduced in technical replicates but is difficult for biological replicates.** A. Scatterplot of technical replicates of synergy score based on Loewe additivity model including 16 drug combinations on three GBM cultures. Replicate 1 versus replicate 2 are significantly similar (p<0.0001). B. Scatterplot of Biological replicates of synergy score based on Loewe additivity model including 16 drug combinations on three GBM cultures. P-value was calculated using the regression coefficient and a number of sampling (n=48), resulting in a t-factor. See Supplementary Tables 11 and 13 for source data.


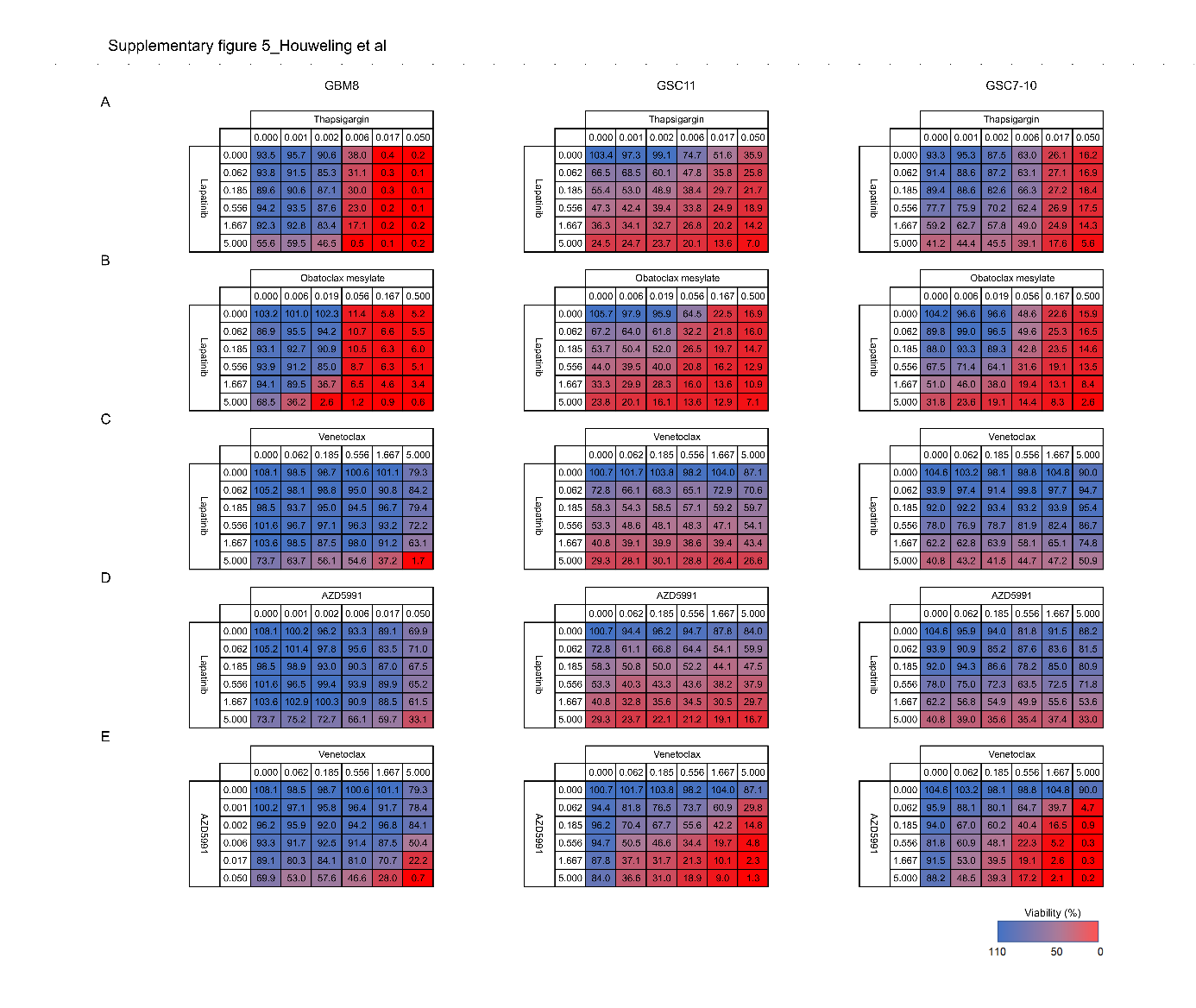


**Supplementary Figure 5. Viability heatmaps of the dual combinations including Lapatinib, Thapsigargin, Obatoclax Mesylate, Venetoclax and AZD5991.** A-E. Viability heatmaps of dual drug combinations in GBM8 (PDGFRA amplified), GSC11 (EGFR amplified) and GSC7-10 (EGFR gain), showed a strong effect on viability when combining Lapatinib with Obatoclax or Lapatinib with Thapsigargin in all three GBM cultures and a strong interaction effect as well when combining Venetoclax and AZD5991 in GSC11 and GSC7-10. Relative light units were normalized via DMSO control; cell viability is shown as mean (n=3, see legend).


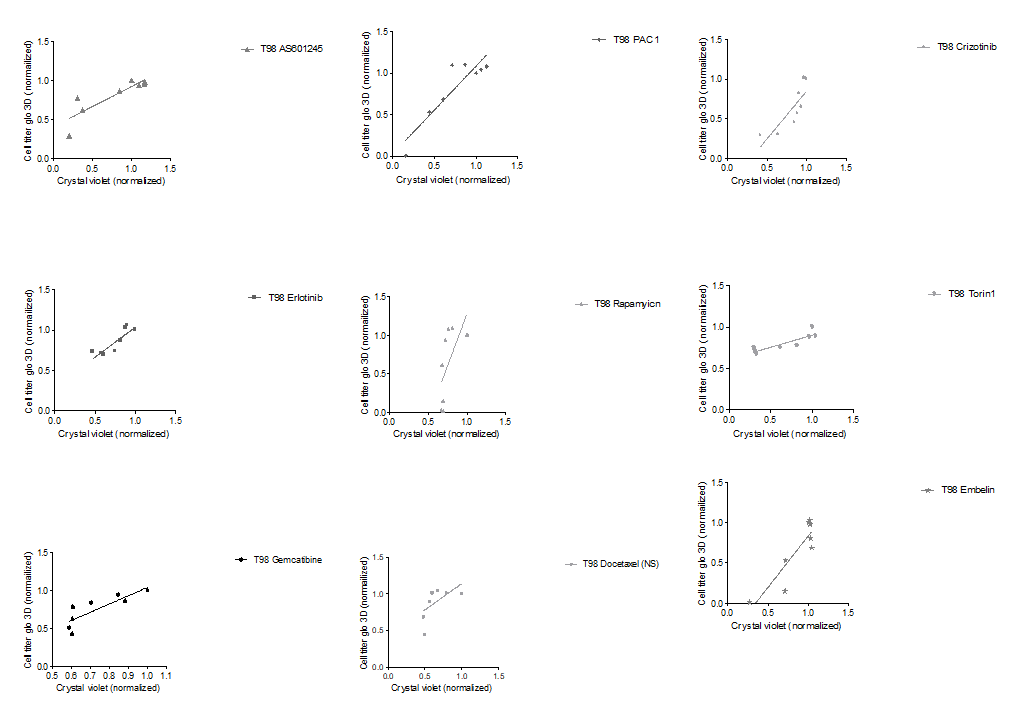

**Supplementary Figure 6. Viability measurements based on crystal violet (total protein/DNA staining) match with cell titer glo (ATP based) measurement in most cases.**
